# Teaching and Researching Motivation

**DOI:** 10.3389/fpsyg.2021.804304

**Published:** 2021-12-16

**Authors:** Yongkang Yuan, Hongjie Zhen

**Affiliations:** ^1^School of Languages and Culture, Tianjin University of Technology, Tianjin, China; ^2^Department of Maritime, Hebei Jiaotong Vocational & Technical College, Shijiazhuang, China

**Keywords:** language teaching, students motivation, language learning (L2) motivation, motivation research, teacher motivation to work

The third edition of *Teaching and Researching Motivation* offers newly-updated and extended coverage of motivation research and pedagogical practice. As in the 2001 and 2011 editions, the text provides comprehensive insights into motivation research and teaching. However, the current edition, as in the authors' words, is “not so much a revised version as a newly written book that has the same authors, the same title and the same structure as the previous one” (Dörnyei and Ushioda, 2021, p. x). It reflects the dramatic changes in the field of motivation research and examines how theoretical insights can be used in everyday teaching practice.

The monograph comprises four parts. Part I, “What is Motivation?”, consists of four chapters. The first chapter pertains to the complex meaning of the term “motivation” and summarizes the key challenges of theorizing motivation. Appealing to us in this chapter is that the authors put a stronger emphasis on understanding motivation in relation to learning LOTEs (languages other than English) and in relation to individual multilingualism. It is altogether fitting and proper for them to hold this belief since the world is becoming more diversified in terms of multilingualism. Chapter 2 offers a historical overview of the most influential cognitive motivation theories. In the new edition, social cultural factors impacting students' motivation are elaborated in more detail. Chapter 3 presents a historical overview of theories of L2 motivation. Drawing on insights from L2 research and psychology, Dörnyei and Ushioda articulate nine interrelated conditions for the motivating capacity of future L2 self-image. With a focus on the L2 Motivation Self System theory, Chapter 4 also critically examines other new theoretical approaches emerged in the field of L2 motivation over the past decade. Finally, it highlights two new perspectives: a focus on L2 learner engagement and “small lens” approaches.

Part II, “Motivation and Language Teaching,” includes three chapters on issues related to the relation between motivation and language teaching. Chapter 5 explores the extent to which theoretical and research insights can lead to practical recommendations for motivating the students in and outside of the language classroom. Based on this principle, it presents instructive approaches to motivating language learners. It also eloquently holds that motivational self-regulation and learner autonomy are two potent energizers which will have a lasing impact beyond the classroom. Chapter 6, “Motivation in Context,” deals with the “dark side” of motivation, “demotivation.” It argues that focused interventions can have significant positive outcomes and help counteract demotivation and facilitate remotivation within second language acquisition (SLA). The last chapter in this part is of special interest as it explores the relationship between language teacher and learner motivation, highlighting possible self theory (exploring conceptual change in language teachers). As a Chinese idiom goes, teaching benefits teachers and students alike. The same is true of language teacher motivation. It argues that teachers' passion and enthusiasm facilitate their teaching and enhance students' learning; and vice versa.

Important and of significance is how to do research so that it can facilitate teaching. Part III, “Researching Motivation,” includes two chapters on issues related to primary, data-based motivation research. Chapter 8 covers the unique characteristics, challenges and research strategies that are specific to the empirical study of language learning motivation. An outstanding contribution of this chapter is that four insightful principles of designing L2 motivation studies are proposed. Followed up on an overview of the most useful methods in this field in the past, Chapter 9 examines two new research initiatives: adopting a complex dynamic system approach and researching unconscious motivation, which will hold particular promise for the future.

Part IV, “Resources and Further Information” is informative and inspiring. In Chapter 10, the authors judiciously remark that particular aspects and context of L2 learning as well as multilingual communication should be focused in the future after further elaborating the interdisciplinary nature and challenge of L2 motivation research. The last chapter contains lists of key sources and resources on motivation such as relevant journals and latest valuable collections, database, discussion groups, and networks. What is of particular value is key scholars of L2 language motivation research, as well as useful tools and measures for researching motivation.

This monograph is a thought-provoking book. Firstly, this new edition reflects the latest research advancement, providing the language teachers and researchers with insights into cultivating motivation. In terms of theoretical paradigm, the L2 Motivation Self System (L2MSS) introduces a holistic approach exploring the combined and interactive operation of a number of different factors in relation to L2 motivation rather than the traditional cause-effect relation between isolated variables. Two recent motivational paradigms originate in L2MSS: *directed motivational currents* and *long-term motivation*, focusing on not only what generates language learning motivation but also on what can sustain motivation long enough. In terms of research method, integration of quantitative and qualitative method (e.g., *questionnaire* + *interview*) has almost become a new trend in the L2 motivation field.

Secondly, Dörnyei and Ushioda provide ideas for theoretical and empirical research by reviewing studies made by them and other researchers. Although a great deal of knowledge has been accumulated, Dörnyei and Ushioda particularly point out two under-explored topics: unconscious motivation and language learner engagement. They also recommend two cutting-edge approaches: “small lens” approaches (actual cognitive process in the mastery of an L2) and complex dynamic systems approach.

Lastly, it is a valuable guide for L2 teachers and researchers. Chapter 5 presents strategies and approaches to motivating language learners such as promoting student engagement and applying technology. Particularly, the up-to-date and rich selection of empirical studies in Chapter 9 are vivid illustration of the research methods, showing language teachers templates of doing research by teaching. The research interests of important scholars listed in Chapter 11 allow language teachers and researchers to follow the current significant research areas on L2 motivation.

Nevertheless, there are still some aspects for this book to be improved in the next edition. First, readers may hope to find a detailed discussion of the social cultural factors impacting teachers' motivation. Second, the key researchers listed in Chapter 11 are mainly in the English world. Had the authors included more key researchers in the non-English world, it would have been more insightful.

All in all, with this new edition, Dörnyei and Ushioda make a very important contribution to our radically new understanding of teaching and researching motivation. As a clear and comprehensive theory-driven account of motivation, this volume can be applied in many different ways. It can be used as a reference book for teachers and/or researchers to review and reflect on motivation teaching and research practice. In addition, it is also of significance in pre-service and in-service teacher education programme. Graduates in applied linguistics, education and psychology can gain plenty of insights from the research findings and additional information offered in this volume. Therefore, this volume is an invaluable resource for teachers and researchers alike.

## Author Contributions

YY: drafts and revision. HZ: revision and supervision. All authors contributed to the article and approved the submitted version.

## Funding

This opinion was supported by the Project of Teaching Reform at Tianjin University of Technology (Grant No: KG20-08).

## Conflict of Interest

The authors declare that the research was conducted in the absence of any commercial or financial relationships that could be construed as a potential conflict of interest.

## Publisher's Note

All claims expressed in this article are solely those of the authors and do not necessarily represent those of their affiliated organizations, or those of the publisher, the editors and the reviewers. Any product that may be evaluated in this article, or claim that may be made by its manufacturer, is not guaranteed or endorsed by the publisher.
